# Assessment of Knowledge of Celiac Disease and Associated Conditions Among Dietitians in Jordan

**DOI:** 10.3390/ijerph22030442

**Published:** 2025-03-17

**Authors:** Hala K. Nawaiseh, Lisako J. McKyer, Dana N. Abdelrahim, Hayder A. Al-Domi, Furat K. AL-Nawaiseh, Mohammad S. AL-Assaf, Shatha A. Abu AL-Nadi

**Affiliations:** 1 Department of Nutrition and Food Technology, School of Agriculture, The University of Jordan, Amman 11942, Jordan; h.aldomi@ju.edu.jo (H.A.A.-D.); sda8190138@ju.edu.jo (S.A.A.A.-N.); 2 Department of Health Promotion and Community Health Sciences, Texas A&M School of Public Health, College Station, TX 76107, USA; lisako.mckyer@unthsc.edu; 3 Research Institute for Medical and Health Sciences, Sharjah University, Sharjah 27272, United Arab Emirates; dabdel-rahim@sharjah.ac.ae; 4 Jordan Center for Disease Control (JCDC), Amman 11941, Jordan; f.nawaiseh@jcdc.gov.jo; 5 Department of Ears, Nose and Throat, King Hussein Medical Centre (KHMC), Amman 11941, Jordan; mohammadalassaf@yahoo.com

**Keywords:** celiac disease, nutritionist, knowledge, management, Jordan

## Abstract

Background: Celiac disease (CD) is a type of systemic autoimmune condition triggered by gluten consumption among genetically predisposed individuals. Aim: To assess the knowledge, awareness, and practices pertaining to CD and associated conditions among dietitians in Jordan. Method: A cross-sectional web-based survey was carried out between April and October 2023. The survey was an internet-based questionnaire with closed-ended questions. Results: The majority of dietitians answered correctly that CD is caused due to an immunological reaction to gluten, gliadin, and protamine (91.7%); it is an autoimmune disease (71.2%); and the risk of developing an autoimmune disease is higher among CD patients (78.8). The majority of respondents (93.6%) correctly identified that a strict gluten-free diet is the treatment approach for CD patients. However, only (18.9%) of dietitians correctly identified the FDA guidelines for “Gluten Free” food labeling. Approximately 53.4% of respondents identified immunoglobulin (IgA) antibody testing as the most reliable way to diagnose patients with CD. Conclusions: The dietitians have a good understanding of CD topics. The development of credentials in CD would ensure that dietitians practicing in CD are skilled.

## 1. Introduction

Gluten-related disorders can be classified into three categories according to their pathogenesis: autoimmune (celiac disease (CD)), allergic (IgE- or non-IgE-mediated wheat allergy (WA)), and non-autoimmune/non-allergic (non-celiac gluten sensitivity [NGCS]) [1]. Celiac disease (CD) is a systemic immune-mediated disease triggered by gluten consumption among genetically predisposed individuals [2]. However, non-celiac gluten sensitivity is a condition in which gastrointestinal and extra-intestinal symptoms are triggered by gluten consumption in the absence of celiac-specific antibodies and villous atrophy, as well as of any allergy-related mechanisms [3]. On the other hand, depending on the route of allergen exposure, WAs are classified into occupational asthma (baker’s asthma) and rhinitis; food allergies (FAs), affecting the skin, the gastrointestinal tract, or the respiratory tract; or wheat-dependent exercise-induced anaphylaxis (WDEIA) and contact urticaria [4]. Gliadin and glutenin are members of the prolamin and glutelin family of proline-rich proteins (PRPs), which are found in wheat. Gliadin and homologous prolamins (secalin in rye, hordein in barley, and avenins in oats) are collectively known as gluten [5,6]. With a rising prevalence of CD as high as 1–2%, it is one of the most prevalent chronic diseases [7]. It has been reported that the incidence of CD in the Middle East is high among both at-risk groups and the general population; however, this is due to dietary eating behaviors such as an excessive consumption of barley and wheat, as well as due to a higher frequency of DR3-DQ2 haplotypes [8].

The HLA class II molecules HLA-DR and HLA-DQ are heterodimeric trans-membrane glycoproteins composed of α and β chains, expressed mainly on professional antigen-presenting cells. They act as receptors of processed self and non-self peptides, which are presented to CD4 + T lymphocytes [9]. Data have indicated that DR3- DQ2 is found in about 90% of CD and 55% of type 1 diabetes mellitus (T1DM) patients; however, DR4-DQ8 is found in about 10% of CD and 70% of T1DM patients [10]. The ability of HLA-DR and HLA-DQ molecules to bind peptides from insulin-secreting cells of the pancreas or sets of gluten-derived peptides, respectively, is considered the primary mechanism of their involvement in T1DM and CD pathogenesis [11]. Moreover, the risk of CD differs between these two haplotypes: the presence of DR3–DQ2 is considered to confer a higher risk than the presence of DR4–DQ8 [11]. The HLA class II genes DQ2 and DQ8 are the main indicators of a shared genetic background between T1DM and CD, and tend to be a significant risk factor for both conditions [12]. Data have indicated that DQ2 and DQ8 confer a 30–50% risk of developing T1DM [12]. According to the American Diabetes Association, an early screening for CD among T1DM-diagnosed children is highly recommended—the earlier the better, as most CD diagnoses are being made within the first years of T1DM diagnosis [12]. Recent studies have reported CD prevalence similar to European countries in some Middle East countries (particularly Iran) [13]. Data have indicated that the prevalence of CD in Arab countries ranges from 0.14% to 3.2% among the healthy adult population [14]. However, in Egypt, a serologic screening conducted among the pediatric general population (n = 1500, age range 7 months to 18 years) demonstrated a prevalence of 0.53% to 6.4% in children with TIDM [15]. In Jordan, data from a previous Jordanian study in 1996 show that the incidence of CD was 1:2800 live births, with a prevalence of 7:100,000 [16]. It has been estimated that up to 80% of CD cases remain undiagnosed [17]. On the other hand, there are still few data, mostly from small sample sizes, about diagnostic delays in children, as well as potential risk factors [18]. There have been numerous reports of diagnostic delays, some of which have lasted up to 10 years [18].

Healthcare providers (HCPs) are aware of the variety of symptoms that CD can present with, which has made them aware of potential diagnosis methods. Epidemiological studies have suggested that for each diagnosed CD case, there could be 3–7 undiagnosed cases [19]. As a primary intestinal disease, CD can cause serious harm to the intestinal mucosa, with malabsorption and dietary deficits as a result [7]. It has been found that untreated CD may lead to growth failure, iron deficiency, vitamin B12 anemia, and osteopenia [7]. Patients with autoimmune hepatitis (AIH), inflammatory bowel disease, Down syndrome, and type I diabetes mellitus have been found to have a higher risk of CD [20].

Also, data have indicated that the prevalence of CD in patients with chronic autoimmune thyroiditis (CAIT) is estimated to be between 2 and 7.8% [21]. Also, children with selective immunoglobulin A (IgA) deficiency have a 10–20-fold increased risk of CD [20]. Also, CD has been found to have a 7.5% pooled prevalence among first-degree relatives [22].

A multidisciplinary Task Force of physicians proposed the “Oslo classification” of CD in 2013, which provides standard definitions for terms associated with CD. Classical CD is characterized by signs and symptoms of malabsorption, diarrhea, loss of appetite, abdominal distention, failure to thrive, bloating, abdominal pain, and muscle wasting [22]. However, the non-classic type comprises CD without signs and symptoms of malabsorption, chronic constipation not responding to usual treatment, chronic abdominal pain, iron-deficient anemia resistant to oral iron supplements, short stature, delayed puberty, abnormal liver biochemistry, arthritis osteopenia/osteoporosis, irritability, chronic fatigue, neuropathy, and unexplained infertility [22].

The first step in CD diagnosis is a screening test, which can be carried out in two cases: firstly, when there is clinical suspicion of CD, i.e., a presence of specific symptoms, including recurrent, chronic diarrhea, constipation, feeling of abdominal distension, chronic iron anemia, and osteoporosis [23]; secondly, when a patient belongs to a risk group, such as first-degree relatives with CD, history of autoimmune diseases, Down syndrome, Turner syndrome, Williams–Beuren syndrome, or IgA deficiency [23]. Moreover, the screening test involves the simultaneous determination of two laboratory parameters: firstly, total IgA antibody level (however, this is only performed to verify if the test has diagnostic value); secondly, the level of antibodies relative to tissue transglutaminase (TGA) in the IgA class [23]. Delays in diagnosis have been associated with the development of systemic complications, including growth failure, delayed puberty in young children, infertility, and enteropathy-associated T-cell lymphoma [24,25]. A potential cause of long diagnostic delays could be a lack of awareness among HCPs about CD, including dietitians [26]. It is well known that it is very important to adhere to a strict gluten-free diet (GFD) to avoid long-term complications associated with CD [27,28]. According to Codex Alimentarius recommendations, for products referred to as gluten-free food, the gluten content should not exceed 20 mg/kg (ppm) in the food as sold or distributed to the consumer [29,30]. Meeting with a skilled registered dietitian nutritionist (RDN) has been identified as an essential aspect of managing CD [31]. Also, in order to ensure appropriate management and avoid complications associated with CD, healthcare professionals play a critical role in both management and follow-up among patients with CD [32]. Both physicians and gastroenterology nurses play a vital role in enhancing adherence to the GFD through education and community engagement [32]. Collaboration between dietitians, gastroenterologists, nurses, and other specialists is vital to assess symptom resolution and provide comprehensive care among patients with CD [33] However, a lack of knowledge about CD might influence the adherence of patients to the GFD [26]. RDNs are nutrition experts; while managing patients with CD may be a specialty, every RDN should know how to use scientifically based information to manage CD [34]. It is well known that dietitians in the educational field must understand the importance of carefully considered allergy education [34]. One of the key components of early CD diagnosis is disease awareness. Understanding the variety of CD symptoms and signs, as well as dietitians’ high levels of suspicion, are essential for early diagnosis. The only treatment for CD is a lifelong adherence to a strict GFD [35]. However, implementing and maintaining a GFD can be challenging among CD patients due to the pervasive nature of gluten in many food items [36]. Several studies have indicated that HCPs, including dietitians, often have inadequate knowledge about CD, contributing to poor disease recognition, diagnostic delays, and ineffective management [26,36]. This has highlighted the urgent need to improve the education and awareness initiatives within the healthcare community, including dietitians. There are limited studies investigating the knowledge regarding CD among dietitians in Jordan. This study is important and can serve as an important driver of raising awareness campaigns in Jordan and in the Middle East.

## 2. Materials and Methods

### 2.1. Research Aims

The aim of this research was to assess the knowledge, awareness, and practices pertaining to CD and associated conditions among dietitians in Jordan.

### 2.2. Research Design

The descriptive design of this study was a cross-sectional web-based survey carried out between June and October 2023. The survey was an internet-based questionnaire (Supplementary Materials) using a Google form with closed-ended questions in both English and Arabic languages.

### 2.3. Sample Population and Piloting

This study was conducted using a non-probability convenient sample of 264 dietitians working in Jordan. The inclusion criteria for this study were adults (over 18 years); having a BSc degree at least in clinical nutrition and dietetics or any higher educational level; either sex; residents in Jordan; Jordanian or any other Arab nationality; willing to participate; and agreed to and signed the consent form to participate. The exclusion criteria were non-nutrition/dietetics specialties, and those not residing in Jordan and not working in Jordan. Participants who met the inclusion criteria were invited to complete the online questionnaire, while those who did not meet the criteria were excluded from participation. The questionnaire was pilot-tested on a sample of 50 adults for validity to assure the simplicity and clarity of the questionnaire. This was to make sure that the questionnaire was well formulated and helped in checking the understandability before the start of the data collection step via the electronic questionnaire.

### 2.4. Data Collection Questionnaire Tool and Its Scoring

The self-administered valid online questionnaire was adopted from a previously validated questionnaire [7,17] to assess the knowledge of the nutrition specialists. The self-reported questionnaire was distributed by social media platforms to a large audience. The questionnaire was divided into two sections: The first section comprised basic demographic information, including sex, age, residency, job sector, years of experience in dietetics, and primary out-patient focus, in addition to some information about whether the dietitian is a member of the Jordanian celiac society or not and the number of newly diagnosed CD cases and follow-up cases in their clinics in the past 12 months. The second section included questions on 3 major domains of knowledge, including causes and complications (6 questions), diagnosis (5 questions), and the nutritional management of CD (8 questions).

Items were in multiple-choice format with a single best correct answer, incorrect answers, and the choice of “I don’t know” to prevent participants from selecting the correct answer by chance. Each correct answer was given a score of one (1). The wrong and the “I don’t know” options were scored as incorrect and given a “zero mark”. Data were analyzed quantitively by calculating the percentage of participants who answered that they were aware of each characteristic known to be associated with CD. The summation of the scores of the correct answers was undertaken to generate a score for each domain and the overall scores. The maximum general score was 19. The overall knowledge scores were grouped according to sex, age, residency, job sector (governmental, private, online, private clinic), years of experience in dietetics, and primary out-patient focus, in addition to some information about whether the dietitian is a member of the Jordanian celiac society or not and the number of newly diagnosed CD cases and follow-up cases in their clinics in the past 12 months. The average score of each answer for each question within each group of nutritionists was calculated.

### 2.5. Statistical Analysis

The socio-demographic data were described as categorical variables using frequencies and percentages of observed values. The data were checked for normality. Significance was analyzed using a Chi-Square test. Continuous variables were summarized by the mean and standard deviation (SD). The differences between the two categories were analyzed using independent *t*-tests, and the differences between more than two categories were analyzed using ANOVA followed by LSD post hoc analysis. Data analysis was conducted using IBM SPSS Version 28.0 (USA). All data significance levels were set at *p* < 0.05.

## 3. Results

The characteristics of the study sample can be described as follows: almost all of the participants were female (97%) and aged between 22 and 31 years (80.7%). Over two-thirds of the participants had a bachelor’s degree (73.8%). The participants were distributed over all job sectors: 11.7% governmental, 30.7% private, 15.2% online, 4.2% personal clinical, and 38.3% other job sectors. One-third of the participants were recent graduates (33.3%), almost one-third had 1–2 years of experience, and the remaining had more than 2 years of experience. The majority of the participants were Jordanian (92%), and the majority of them were working as general dietitians (82.6%). Approximately half of the participants were not members of the Celiac Disease Society (54.9%), while 36.4% had not heard about the society before (Table 1).

Table 2 describes the knowledge degree of the dietitians regarding the causes and complications of celiac disease. For the first four questions, the number of participants who chose the correct answer was significantly higher than the participants who chose the wrong or unsure answer choices. The percentages of participants who chose the correct answers to the first four questions—“CD is caused due to an immunological reaction to?”, “CD is an autoimmune disease?”, “Risk of developing an autoimmune disease is higher among CD patients?”, and “The prevalence of CD in patients with T1DM is higher than in the general population?”—were 91.7%, 71.2%, 78.8%, and 56.8%, respectively. For the sixth question, “Dietitians focus on nutritional assessment for patients with CD?”, there was confusion about how to assess patients with celiac disease; the participants mainly preferred the choice of “sources of gluten in the diet”, but there was not a significant distribution (*p* = 0.066).

The part regarding the dietitians’ knowledge about the diagnosis of celiac disease consisted of five questions: “The most reliable test (100%) in CD diagnosis is?”, “Delays in CD diagnosis can lead to nutritional deficiencies?”, “The classical symptoms of CD include abdominal pain, diarrhea and weight loss?”, “The non-classical symptoms of CD include extreme weakness, anemia, oral ulcers, and infertility?” and “Many individuals with CD are lactose-intolerant when they are newly diagnosed?”. The percentages of participants who chose the correct answers were 26.9%, 80.7%, 92.8%, 72.7%, and 61.0%, respectively, and all of the responses to these questions were at a *p*-value less than 0.05 (Table 3).

Table 4 explains the distribution of answers to the questions about the nutritional management of celiac disease. This section consisted of eight questions. Out of the eight questions, six received correct answers from a higher percent of participants: “The treatment approach for CD consists of?”, “CD patients should completely avoid the following”, “The gluten-free diet should be based on gluten-free whole grains such as”, “Patients with CD should be encouraged to consume gluten-free whole grains over gluten-free refined grains such as white rice, and milled corn?”, “Iron supplementation in gluten-free multivitamins may be required for CD patients”, and “Patients with CD are encouraged to drink calcium-rich food”. The percentages of correct answers were 93.6%, 89.8%, 72.7%, 78.0%, 83.3%, and 72.7% respectively, and the distribution in all of these questions was significant (*p*-value ˂ 0.05). Unfortunately, in two questions—“Are dried potatoes, corn oil, sea salt, sucrose, fructose, tomato paste, maltodextrins, and citric acid unsafe ingredients in the CD celiac diet labeling” and “FDA guidelines for Gluten-Free food labeling”—the higher percentages with a significant distribution did not reflect the correct answer. The percentages of correct answers were 42.4% and 18.9% (*p*-value ˂ 0.05).

The mean total score of the first section, “knowledge about causes and complications”, was 4.03 ± (SD = 1.29) out of 6 marks. For the second section, “knowledge about diagnosis”, the mean score was 3.34 (SD = 1.14) out of 5 marks, and in the third section, “knowledge about management”, the mean score was 5.48 (SD = 1.48) out of 8 marks. The total knowledge score was 12.86 ± 2.99 out of 19 marks (Table 5). We categorized the knowledge among the current participants regarding causes and complications, diagnosis, and management based on mean ± S.D into high, intermediate, and low. The following results were found for knowledge of causes and complications: high knowledge: 5.33–6; intermediate: 2.75–5.32; low: 0–2.74. On the other hand, the diagnosis knowledge categories were as follows: high knowledge: 4.49–5; intermediate: 2.21–4.48; low: 0–2.20. The management knowledge categories were as follows: high knowledge: 6.96–8; intermediate knowledge: 4.00–6.95; low knowledge: 0–3.99. The current findings indicate that the dietitians had intermediate knowledge in all three domains: causes and complications, diagnosis, and management.

In addition to this, we compared the total knowledge score with the sociodemographic data of the participants (Table 6). Female participants showed better total knowledge (12.91 ± 2.93) than male participants (11.25 ± 4.46) (*p* = 0.037). Older dietitians (≥42 years) (14.21 ± 4.34) had significantly higher total knowledge scores than the middle-aged group (32–41 years) (12.92 ± 3.38) and the youngest dietitians (22–31 years) (12.76 ± 2.81) (*p* = 0.0001). The PhD, and MSc, education-degree-holder participants showed significantly (*p* = 0.0001) higher total knowledge scores than the BSc-holder participants; the total knowledge scores for the degrees were 13.89 ± 3.98, 13.27 ± 2.79, and 12.78 ± 2.86, respectively. The more years of experience the dietitians had, the higher the total knowledge score (*p* = 0.002), starting from 14.05 ± 3.71 for those who had 10 years of experience or more, and dropping to 12.09 ± 2.86 for freshly graduated dietitians. There were no significant differences between dietitians’ job sectors, residencies, primary outpatient focus, membership status regarding the Celiac Disease Society, or number of newly diagnosed cases/follow-up cases.

## 4. Discussion

The current findings indicate that the majority of dietitians in Jordan have intermediate knowledge related to the causes and complications, diagnosis, and management of CD. Our findings show the majority of dietitians in Jordan answered correctly that CD is caused due to an immunological reaction to gluten, gliadin, and prolamin (91.7%); it is an autoimmune disease (71.2%); the risk of developing an autoimmune disease is higher among CD patients (78.8%); the prevalence of CD in patients with type 1 diabetes mellitus (T1DM) is higher than in the general population (56.8%); and they should focus mainly on the source of gluten in the diet (62.5%). The current findings highlight a strong understanding of the critical aspects of CD among dietitians in Jordan—particularly its causes and complications, diagnosis, and management. However, the development of credentials in CD would ensure that dietitians practicing in CD are highly skilled.

Registered dietitian nutritionists (RDNs) must be equipped with sufficient knowledge about CD and the GFD in order to provide optimal nutritional care to patients with CD and non-celiac gluten sensitivity [37]. CD may be a specialty area; however, every RDN should know how to use scientifically based information to manage it [34]. Similar to the current findings, it has been found that a high percentage (65%) of RDNs have reported a high level of knowledge regarding CD definition and a low percentage (30%) of RDNs have reported a high level of understanding of local CD support groups [38]. The 2004 National Institutes of Health (NIH) Consensus Development Conference on CD recommended the involvement of dietitians in CD management [39]. There is evidence that when asked to choose among several referral options, patients have shown a preference for dietetic follow-up [40]. A survey among patients with CD reported that only 40% of patients agreed with the statement “It is hard to find a dietitian knowledgeable about GFD” [31]. Moreover, 79% of them reported having seen a dietitian, but 39% had only seen a dietitian once [31]. Based on the Academy of Nutrition and Dietetics Evidence Analysis Library, medical nutrition therapy provided by a registered dietitian is strongly recommended among individuals with CD [41]. Adherence to the GFD is vital in the management of CD; however, patients with CD must be monitored by an experienced dietitian to assess the effectiveness of the GFD, quality of life, symptom improvement, and barriers to compliance [42]. It has been found that the role of dietitians specializing in digestive diseases is crucial, not only to establish appropriate dietary guidelines related to CD, but also to provide reliable dietary education, monitor ongoing treatment [43], and help in detecting unintentional gluten ingestions [42]

Several studies have provided opinions of individuals with CD about registered dietitians [44,45]. The respondents rated the quality of information received from dietitians as excellent (26%), very good (28%), good (22%), and fair/poor (24%) [44]. However, in another survey, only 21% of CD patients who referred to a dietitian found that the information obtained was helpful [45]. Several studies have indicated that poor knowledge among HCPs, including dietitians, leads to the disease being underdiagnosed, adding to the long diagnostic delays of the disease [46]. In similar studies among HCPs to assess their nutritional knowledge related to CD, approximately half of respondents rated their knowledge as sufficient [47,48]. Nonetheless, according to survey data collected from 160 CD patients, 54% of participants stated that RDNs did not appear to be knowledgeable about CD, and 53% of patients did not believe that RDNs were beneficial [24]. Most RDNs are able to identify CD and non-celiac gluten sensitivity (NCGS) as conditions requiring a GFD [37]. However, it is alarming that approximately 50% of RDNs would recommend a GFD for those with a wheat allergy [37]. While the prevalence of CD in the general population is estimated to be between 0.3 and 1%, it is significantly higher among patients with T1DM as a result of genetic predisposition [49]. Therefore, screening for CD is recommended for all patients with T1DM [50]. Also, international guidelines have proposed CD screening for newly diagnosed T1DM patients and follow-up in the first 5 years [51].

Our findings indicated that the majority of respondents (93.6%) correctly identified that a strict gluten-free diet is the appropriate treatment approach for CD patients. Also, the majority of dietitians correctly recognized that iron supplementation in gluten-free multivitamins may be required for CD patients (83.3%), and patients with CD are encouraged to drink calcium-rich food (72.7%). However, only 18.9% of dietitians correctly identified that FDA guidelines for “Gluten Free” food labeling are limited to 20 ppm. Following a GFD can be difficult for patients with CD because it is linked to a diet that can cause nutritional deficiencies or over-sufficiency [52]. It has been reported that there is a misconception by the public that gluten is a carbohydrate, and it has been indicated that RDNs may also believe that gluten is a carbohydrate [37]. Gluten consumption in CD patients triggers both innate and adaptive immune responses, leading to small intestinal mucosal crypt hyperplasia and villous atrophy [53,54]. These alterations result in nutrient malabsorption, which can cause deficiencies in a number of fat-soluble vitamins, minerals, and micronutrients, such as iron, folic acid, copper, zinc, vitamin B6, vitamin B12, vitamin D, vitamin E, and vitamin K [53,54]. Iron is mainly absorbed in the proximal small intestine, the region most affected by CD [53,54]. It has been found that another possible cause of iron deficiency is the loss of duodenal enterocytes, the storage site of ferritin [55]. Iron deficiency anemia is one of the most common clinical manifestations, affecting up to 32% of CD adults [46]. If a deficiency is detected, supplementation should be initiated and maintained until remission of the symptoms [55]. Also, it has been found that about 30–60% of newly diagnosed patients with CD show decreased bone mineral density (BMD) and 18–35% present with osteoporosis [56,57]. The multifactorial mechanism of osteoporosis among CD patients includes systemic and local inflammation associated with CD, vitamin D deficiency, and impaired calcium absorption, which results in secondary hyperparathyroidism and increased bone resorption [58]. Also, mucosal damage among patients with CD impairs calcium absorption, leading to impaired bone health [59]. Primarily, lactose intolerance is related to an impaired release of lactase enzymes because of damaged mucosa, which may further limit dairy intake [59]. Data have indicated that pediatric patients with untreated CD are at risk of short stature and a constitutional delay of puberty [60].

Similar to the current findings, an online survey of HCPs, including dietitians, revealed that dietitians had the highest awareness of the risk of nutritional deficiencies among CD patients (82%) compared to physicians (52%), nurses (52%), and medical students (54%) [61]. Moreover, only 10% of HCPs rated their knowledge of the GFD as sufficient; the largest proportions of these were dieticians (35%) and dietetics students (10%) [61].

However, the majority of respondents (93%) recommended that a dietitian should be consulted before introducing the GFD [7]. Furthermore, a previous study discovered that the majority of doctors (75%) believed that a lifelong GFD was the only pathogenetic treatment for CD; however, the reset did not work well enough to treat CD patients with a dairy-free diet, a temporary GFD, or H. pylori eradication therapy [8]. The identification and treatment of nutritional deficiencies has been identified as one of the essential aspects of managing CD [62]. Healthcare professionals should encourage patients with CD to learn about healthy diets.

There are several recommendations, including the following: avoiding highly processed foods that are low in fiber and high in sugars and poor-quality fats; foods with a low glycemic index and high-quality fats are recommended; the consumption of whole-grain gluten-free flours, including pseudocereals and pulses, is recommended; homemade gluten-free foods should be encouraged; and the consumption of fruits and vegetables should be promoted [63]. The current data found that the majority of dietitians (53.4%) identified immunoglobulin (IgA) antibody testing as the most reliable way to diagnose patients with CD, followed with a biopsy of twelve tissues (26.9%). The 2013 CD guidelines from the American College of Gastroenterology (ACG) recommend using a combination of small intestinal biopsy and serologic tests for diagnosing CD [64]. Small-bowel mucosal villous atrophy and crypt hyperplasia are considered the gold standard in the diagnosis of CD [65]. However, serological antibody screening tests have long been recognized in preselecting patients to undergo diagnostic small-intestinal biopsy [65]. The most sensitive antibody test for diagnosing CD is the immunoglobulin A (IgA) class endomysia antibody (EMA) assay [66]. Nonetheless, despite a positive serum EMA exhibiting nearly 100% specificity for CD, approximately 10–20% of untreated CD patients show a negative serum EMA [67]. The EMA test is proposed to exhibit reduced sensitivity, particularly among CD children under the age of 2 [65].

Conversely, it has been suggested that EMA may serve as a biomarker for early-stage CD, manifesting in the serum prior to the onset of villous atrophy [68]. However, EMA testing has several disadvantages: it is expensive, subjective, labor-intensive, and requires experienced personnel to perform it [66]. For more than 40 years, the primary test for CD has been small-bowel mucosal biopsy; however, the diagnosis should only be made if the biopsy specimen shows villous atrophy with crypt hyperplasia during a typical gluten-containing diet [69]. However, initial screening for various CD antibodies is still needed to select patients to undergo small-intestinal biopsy [69]. The finding of serum celiac antibodies at the time of diagnosis and their disappearance upon a GFD supports the diagnosis, but seronegative results do not exclude the disease [67]. Also, a small-bowel biopsy performed during gastrointestinal endoscopy is the initial diagnostic procedure when there is a high clinical suspicion of CD because of severe signs and symptoms of malabsorption [65]. However, the diagnosis of CD is even more challenging in cases where villous shortening is patchy, being evident only in restricted areas of the small-bowel mucosa [70]. Also, the majority of respondents correctly identified that delays in CD diagnosis can lead to nutritional deficiencies (80.7%); the classical symptoms of CD include abdominal pain, diarrhea, and weight loss (92.8%); and the non-classical symptoms of CD include extreme weakness, anemia, oral ulcers, and infertility (72.7%).

Previous research has found that only one-third of HCPs in Romania performed a total IgA test on patients who were diagnosed with CD [71]. It has been found that one of the most important reasons for delays in CD diagnosis may be the poor knowledge of HCPs regarding CD [26,72]. Delays in CD diagnosis could be up to 10 years [73]. Due to the lack of clinically obvious symptoms among the majority of CD patients, the diagnosis is often missed or delayed [28,74]. It has been reported that the knowledge of HCPs (from five European countries: Croatia, Hungary, Germany, Italy, and Slovenia) regarding the epidemiology, clinical signs and symptoms, diagnostic procedure, and treatment of CD is unsatisfactory; only 51% of HCPs achieved 50% total scores [26]. The current data indicate that dietitians with MSc and PhD degrees have the highest mean knowledge scores (13.27 ± 2.79 and 13.89 ± 3.98, respectively) compared with dietitians with BSc degrees (12.78 ± 2.86). Moreover, dietitians with more than 2 years of experience have reported higher mean knowledge scores compared with those with (1–2) years of experience (*p*-value = 0.002). However, there were no significant differences regarding the number of follow-up appointments for CD out-patients and mean knowledge scores (*p*-value = 0.799). In contrast to the current data, a cross-sectional study was conducted in Alaska, Colorado, Connecticut, Delaware, Montana, Nebraska, and North Dakota in 2013 to assess RDN self-rated CD knowledge, finding that there was no significant correlation between RDNs’ self-reported knowledge and education level [28]. Also, a significant negative correlation has been reported between RDNs’ self-reported knowledge and the number of CD patients seen per week [34]. Moreover, it has been reported that with age, the knowledge of HCPs about CD improves; HCPs with more than 10 years of work experience have better knowledge and better performance in the diagnosis and treatment of CD [75]. Previous research reported that pediatric gastroenterologists achieved the highest scores related to a higher awareness of the burden of CD [26]. Similarly, one study demonstrated that junior doctors outperformed senior doctors due to their recent exposure to CD as medical students, whereas senior physicians were still being taught about CD as a rare malabsorptive disorder [72].

Strengths and limitations: The current study has several strengths: it is the first to evaluate dietitians’ knowledge of CD in Jordan, and also the inclusion of dietitians from all sectors. However, there were several limitations to this research. First, while CD knowledge and awareness are important among dietitians, some dietitians with limited exposure to CD may have struggled with the survey knowledge questions. Second, there was a lack of sex diversity among the dietitian participants. Finally, a small sample size may limit the generalizability of the current findings.

## 5. Conclusions

In conclusion, the findings in the present study indicate that the majority of dietitians in Jordan have intermediate knowledge related to the causes and complications, diagnosis, and management of CD. It is imperative that dietitians are able to provide CD patients with as much reliable information as possible. The identification and treatment of nutritional deficiencies associated with GFD have been identified as essential issues. These clinical implications represent an opportunity to improve certain aspects of the education that current and future RDNs receive regarding CD, gluten, and the GFD in order to maintain the public image of RDNs as experts in food and nutrition. Currently, in Jordan, there are no credential-recognizing dietitian experts in CD. The development of credentials in CD would ensure that dietitians practicing in CD are skilled. Longitudinal studies to assess dietitian knowledge regarding CD are necessary to assess the impact of specialized training programs on dietitians’ competence and confidence in managing CD patients. Also, future research could examine how the introduction of formal credentials in CD could improve both the clinical care of CD patients and the professional development of dietitians.

## Figures and Tables

**Table 1:** Descriptive characteristics of the study population (n = 264).

Characteristics		N (%)
Sex	Male	8 (3.0%)
	Female	256 (97.0%)
Age	22–31 years	213 (80.7%)
	32–41 years	37 (14.0%)
	≥42 years	14 (5.3%)
Education	BSc	195 (73.8%)
	MSc	60 (22.7%)
	PhD	9 (3.4%)
Job sector	Governmental	31 (11.7%)
	Private	81 (30.7%
	Online	40 (15.2%)
	Personal clinic	11 (4.2%)
	Others	101 (38.3%)
Years of experience in dietetics	Fresh graduate	88 (33.3%)
	1–2 years	87 (33.0%)
	3–4 years	30 (11.4%)
	5–10 years	39 (14.8%)
	≥10 years	20 (7.6%)
Nationality	Jordan	243 (92%)
	Other Arabic countries	21 (8.0%)
Primary out-patient focus	Generalist (general dietitian)	218 (82.6%)
	Specialist (renal, diabetes, etc.)	46 (17.4%)
Member of Jordanian Celiac Society	Yes	23 (8.7%)
	No	145 (54.9%)
	I do not know this society	96 (36.4%)
Number of newly diagnosed celiac disease out-patients in the past 12 months	Zero patients	57 (21.6%)
	1–2 patients	64 (24.2%)
	3–4 patients	53 (20.1%)
	≥5 patients	90 (34.1%)
Number of follow-up appointments for celiac disease out-patients in the past 12 months	Zero patients	68 (25.8%)
	1–2 patients	91 (34.5%)
	3–4 patients	52 (19.7%)
	≥5 patients	53 (20.1%)

**Table 2:** Nutritionists’ knowledge about causes and complications of celiac disease (n = 264).

Questions and Provided Answer Choices		Proportion of Participants Choosing the Answer $	Significance of *p*-Value μ
CD is caused due to an immunological reaction to?	Albumin	3 (1.1%)	0.0001 **
	Globulin	10 (3.8%)	
	Gluten, gliadin, prolamin #	242 (91.7%)	
	I don’t know	9 (3.4%)	
CD is an autoimmune disease?	Yes #	188 (71.2%)	0.016 *
	No	71 (26.9%)	
	I don’t know	5 (1.9%)	
Risk of developing an autoimmune disease is higher among CD patients?	Yes #	208 (78.8%)	0.0001 **
	No	23 (8.7%)	
	I don’t know	33 (12.5%)	
The prevalence of CD in patients with T1DM is higher than in the general population?	Yes #	150 (56.8%)	0.0001 **
	No	36 (13.6%)	
	I don’t know	78 (29.5%)	
CD can occur at any age from early childhood to old age?	Yes #	112 (42.4%)	0.006 **
	No	127 (48.1%)	
	I don’t know	25 (9.5%)	
Dietitians focus on nutritional assessment for patients with CD?	Nutritional adequacy/five food groups	44 (16.7%)	0.066
	Source of gluten in the diet #	165 (62.5%)	
	Nutrients at risk (iron, folate, B12).	43 (16.3%)	
	Fiber intake	3 (1.1%)	
	I don’t know	9 (3.4%)	

# Correct answer; $ number; and (%) μ Significant according to Chi-Square test. * *p* < 0.05; ** *p* < 0.0001.

**Table 3:** Nutritionists’ knowledge about the diagnosis of celiac disease (n = 264).

Questions and Provided Answer Choices		Proportion of Participants Choosing Each Answer *	Significance of *p*-Value **
The most reliable test (100%) in CD diagnosis is?	Immunoglobulin (IgA) antibody	141 (53.4%)	0.0001 **
	Classical symptoms	9 (3.4%)	
	Genetics	18 (6.8%)	
	Biopsy of the twelve tissues #	71 (26.9%)	
	I don’t know	25 (9.5%)	
Delays in CD diagnosis can lead to nutritional deficiencies?	Yes #	213 (80.7%)	0.0001 **
	No	22 (8.3%)	
	I don’t know	29 (11.0%)	
The classical symptoms of CD include abdominal pain, diarrhea and weight loss?	Yes #	245 (92.8%)	0.0001 **
	No	14 (5.3%)	
	I don’t know	5 (1.9%)	
The non-classical symptoms of CD include extreme weakness, anemia, oral ulcers, and infertility?	Yes #	192 (72.7%)	0.0001 **
	No	30 (11.4%)	
	I don’t know	42 (15.9%)	
Many individuals with CD are lactose-intolerant when they are newly diagnosed?	Yes #	161 (61.0%)	0.0001 **
	No	44 (16.7%)	
	I don’t know	59 (22.3%)	

# Correct answer; and (%); μ significant according to Chi-Square test. * *p* < 0.05; ** *p* < 0.0001.

**Table 4:** Nutritionists’ knowledge about the nutritional management of celiac disease (n = 264).

Questions and Provided Answer Choices		Proportion of Participants Choosing Each Answer *	Significance of *p*-Value **
The treatment approach for CD consists of?	Medication	6 (2.3%)	0.0001 **
	Radiation therapy	3 (1.1%)	
	Surgical operation	3 (1.1%)	
	Strict gluten-free diet for life #	247 (93.6%)	
	I don’t know	5 (1.9%)	
CD patients should completely avoid the following:	Barley	9 (3.4%)	0.0001 **
	Pasta	3 (1.1%)	
	Biscuits and snacks made with wheat flour	15 (5.7%)	
	All of the above #	237 (89.8%)	
The gluten-free diet should be based on gluten-free whole grains such as	Whole corn	29 (11.0%)	0.005 **
	Buckwheat	22 (8.3%)	
	Amaranth	6 (2.3%)	
	Quinoa	15 (5.7%)	
	All of the above #	192 (72.7%)	
Patients with CD should be encouraged to consume gluten-free whole grains over gluten-free refined grains such as white rice and milled corn?	Yes #	206 (78.0%)	0.0001 **
	No	41 (15.5%)	
	I don’t know	17 (6.4%)	
Are dried potatoes, corn oil, sea salt, sucrose, fructose, tomato paste, maltodextrins, and citric acid unsafe ingredients in the CD celiac diet labeling	Yes	103 (39.0%)	0.007 **
	No #	112 (42.4%)	
	I don’t know	49 (18.6%)	
FDA guidelines for “Gluten Free” food labeling	5 ppm	20 (7.6%)	0.001 **
	10 ppm	14 (5.3%)	
	20 ppm #	50 (18.9%)	
	0 ppm	24 (9.1%)	
	No FDA guidelines	18 (6.8%)	
	I don’t know	138 (52.3%)	
Iron supplementation in gluten-free multivitamins may be required for CD patients	Yes #	220 (83.3%)	0.0001 **
	No	19 (7.2%)	
	I don’t know	25 (9.5%)	
Patients with CD are encouraged to drink calcium-rich food	Yes #	192 (72.7%)	0.0001 **
	No	23 (8.7%)	
	I don’t know	49 (18.6%)	

# Correct answer; and (%) μ significant according to Chi-Square test. * *p* < 0.05; ** *p* < 0.0001.

**Table 5:** Total scores for the 3 domains and the total knowledge score (n = 264).

Domain Score	Maximum Score	Mean ± S.D.
Causes and complications	6 marks	4.03 ± 1.29
Diagnosis	5 marks	3.34 ± 1.14
Management	8 marks	5.48 ± 1.48
Total knowledge score	19 marks	12.86 ± 2.99

**Table 6:** Mean overall knowledge scores of nutritionists according to their demographic data.

Characteristics		N (%)	Knowledge Score Mean ± SD	*p*-Value
Sex	Male	8 (3.0%)	11.25 ± 4.46	0.037 *
	Female	256 (97.0%)	12.91 ± 2.93	
Age	22–31 years	213 (80.7%)	12.76 ± 2.81 ^a^	0.0001 **
	32–41 years	37 (14.0%)	12.92 ± 3.38 ^a^	
	≥42 years	14 (5.3%)	14.21 ± 4.34 ^b^	
Education	BSc	195 (73.8%)	12.78 ± 2.86 ^a^	0.0001 **
	MSc	60 (22.7%)	13.27 ± 2.79 ^b^	
	PhD	9 (3.4%)	13.89 ± 3.98 ^b^	
Job sector	Governmental	31 (11.7%)	12.74 ± 3.26	0.118
	Private	81 (30.7%	13.75 ± 2.67	
	Online	40 (15.2%)	12.55 ± 3.29	
	Personal clinic	11 (4.2%)	13.82 ± 3.16	
	Others	101 (38.3%)	12.19 ± 2.86	
Years of experience in dietetics	Fresh graduate	88 (33.3%)	12.09 ± 2.86 ^c^	0.002 *
	1–2 years	87 (33.0%)	13.25 ± 2.75 ^b^	
	3–4 years	30 (11.4%)	12.87 ± 3.19 ^b^	
	5–10 years	39 (14.8%)	13.07 ± 2.98 ^b^	
	≥10 years	20 (7.6%)	14.05 ± 3.71 ^a^	
Primary out-patient focus	Generalist (general dietitian)	218 (82.6%)	12.81 ± 2.93	0.255
	Specialist (renal, diabetes, etc.)	46 (17.4%)	13.07 ± 3.31	
Member of Jordanian Celiac Society	Yes	23 (8.7%)	12.04 ± 3.96	0.182
	No	145 (54.9%)	13.27 ± 2.78	
	I don’t know this society	96 (36.4%)	12.43 ± 2.97	
Number of newly diagnosed celiac disease out-patients in the past 12 months	Zero patients	57 (21.6%)	12.47 ± 3.47	0.154
	1–2 patients	64 (24.2%)	12.70 ± 3.19	
	3–4 patients	53 (20.1%)	12.91 ± 2.76	
	≥5 patients	90 (34.1%)	13.18 ± 2.65	
Number of follow-up appointments for celiac disease out-patients in the past 12 months	Zero patients	68 (25.8%)	12.44 ± 2.88	0.799
	1–2 patients	91 (34.5%)	12.98 ± 3.16	
	3–4 patients	52 (19.7%)	12.85 ± 2.89	
	≥5 patients	53 (20.1%)	13.18 ± 2.96	

Number (%); μ significant according to Chi-Square test. * *p* < 0.05; ** *p* < 0.0001. Different letters indicate significant difference.

## Data Availability

The datasets used and/or analyzed during the current study are available from the corresponding author on reasonable request.

## Supplementary Materials

The following supporting information can be downloaded at: https://www.mdpi.com/article/10.3390/ijerph22030442/s1.

## Abbreviations

CD	Celiac disease
WA	Wheat allergy
NGCS	Non-auto-immune/non-allergic (non-celiac gluten sensitivity)
FA	Food allergy
WDEIA	Wheat-dependent exercise-induced anaphylaxis
PRPs	Proline-rich proteins
HCPs	Healthcare providers
AIH	Auto-immune hepatitis
IgA	Immunoglobuline A
TGA	Tissue transglutaminase
GFD	Gluten-free diet
RDN	Registered dietitian nutritionists
TIDM	Type 1 diabetes mellitus
NIH	National Institutes of Health
NCGS	Non-celiac gluten sensitivity
FDA	Food and drug administration
BMD	Bone mineral density
EMA	Endomysia antibody
