# Supplementary material for: Assessment of Knowledge of Celiac Disease and Associated Conditions Among Dietitians in Jordan
Source: Int J Environ Res Public Health. 2025 Mar 17;22(3):442. doi: 10.3390/ijerph22030442 (PMC11941886; doi:10.3390/ijerph22030442)
Supplement: Supplementary file 1 [file ijerph-22-00442-s001.zip › ijerph-3412496-supplementary.pdf]

Supplementary Material

SUPPLEMENTARY APPENDIX 1

Assessment of Knowledge of Celiac Disease and associated conditions among Dietitians in Jordan

Please circle the selected answer:

Demographic Characteristics of Dietitian:

1. **Sex:**

a. Female.
b. Male.

2. **Age:**

a. 22-31 years.
b. 32-41 years.
c. >= 42 years.

3. **Education:**

a. Bachelor
b. Master
c. PhD

4. **Job Sector:**

a. Governmental.
b. Private.
c. Personal Clinic.
d. Online work.
e. I don't work.

5. **Years of Dietetics Experience:**

a. Fresh graduate.
b. 1-2 Years.
c. 3-4 Years.
d. 5-10 Years.
e. > 10 Years.

6. **Residency :**

7. **Primary Outpatient Focus:**
   a. Generalist (General Dietitian).
   b. Specialist (Renal, Diabetes, etc....).

8. **Are you a member of Jordanian Celiac Society?**
   a. Yes.
   b. No.
   c. I don't know this society.

9. **Number of newly diagnosed Celiac Disease outpatient in last 12-month?**
   a. 0
   b. 1-2
   c. 3-4
   d. >5

10. **Number of follow-up appointment with out-patient with CD in last 12-month?**
   a. 0
   b. 1-2
   c. 3-4
   d. >5

Nutritionist's knowledge about prevalence, causes and complications of Celiac Disease

1. **CD is caused due to an immunological reaction to?**

   a. Albumin.
   b. Globulin.
   c. Gluten, gliadin, prolamin.
   d. I don't know

2. **CD is an autoimmune disease?**
   a. Yes.
   b. No.
   c. I don’t know.

3. **Risk of developing an autoimmune disease is higher among the CD patients.**
   a. Yes.
   b. No.
   c. I don’t know.

4. **The prevalence of CD in patients with T1DM is higher than in the general population?**
   a. Yes.
   b. No.
   c. I don't know.

5. **CD can occur at any age from early childhood to old age?**
   a. Yes.
   b. No.
   c. I don't know.

6. **Dietitians focus on nutritional assessment for patients with CD:**
   a. Nutritional adequacy /five food groups.
   b. Source of gluten in the diet.
   c. Nutrients at risk (Iron, Folate, B12).
   d. Fiber Intake.
   e. I don't know.

Nutritionist's knowledge about Celiac Disease Diagnosis:

1. **The most reliable test 100% in CD diagnosis is:**
   a. Immunoglobulin (IgA) antibody.
   b. Classical symptoms.
   c. Genetics.
   d. Biopsy of the twelve tissues.
   e. I don't know.

2. **Delays in CD diagnosis can lead to nutritional deficiencies:**
   a. Yes.
   b. No.
   c. I don't know.

3. **The classical symptoms of CD include abdominal pain, diarrhea and weight loss:**
   a. Yes.
   b. No.
   c. I don't know.

4. **The non-classical symptoms of CD include extreme weakness, anemia, oral ulcers, and infertility:**
   a. Yes.
   b. No.
   c. I don't know.

5. **Many individuals with CD are lactose intolerant when they are newly diagnosed:**
    a. Yes.
    b. No.
    c. I don’t know.

Nutritionist's knowledge about nutritional management of celiac disease

1. **The treatment approach for CD:**

a. Medication.
b. Radiation therapy.
c. Surgical operation.
d. Strict gluten-free diet for life.
e. I don’t know.

2. **CD patients should completely avoid the following:**
a. Barely.
b. Pasta.
c. Biscuits and snacks made with wheat flour.
d. All of the above.

3. **The gluten-free diet should be based on gluten-free whole grains such as:**

a. Whole corn.
b. Buckwheat.
c. Amaranth.
d. Quinoa.
e. All of the above.

4. **Patients with CD should be encouraged to consume gluten-free whole grains over gluten-free refined grains such as (White rice, and milled corn):**

    a. Yes.
    b. No.
    c. I don’t know.

5. **Are dried potatoes, corn oil, sea salt, sucrose, fructose, tomato paste, maltodextrins, and citric acid unsafe ingredients in the CD celiac diet labeling:**

    a. Yes.
    b. No.
    c. I don’t know.

6. FDA guidelines for " Gluten Free" food labeling:

a. 5 ppm.
b. 10 ppm.
c. 20 ppm.
d. 0 ppm.
e. No FDA guidelines.
f. I don’t know.

7. Iron supplementation in gluten-free multivitamins may be required for CD patient:

a. Yes.
b. No.
c. I don’t know.

8. Patients with CD are encouraged to drink calcium-rich food:

a. Yes.
b. No.
c. I don’t know.
